# DIY-Bio – economic, epistemological and ethical implications and ambivalences

**DOI:** 10.1186/s40504-016-0039-1

**Published:** 2016-05-30

**Authors:** Jozef Keulartz, Henk van den Belt

**Affiliations:** Environmental Philosophy at Radboud University, Nijmegen, Netherlands; Wageningen University, Wageningen, Netherlands

## Abstract

Since 2008, we witness the emergence of the Do-It-Yourself Biology movement, a global movement spreading the use of biotechnology beyond traditional academic and industrial institutions and into the lay public. Practitioners include a broad mix of amateurs, enthusiasts, students, and trained scientists. At this moment, the movement counts nearly 50 local groups, mostly in America and Europe, but also increasingly in Asia. Do-It-Yourself Bio represents a direct translation of hacking culture and practicesfrom the realm of computers and software into the realm of genes and cells. Although the movement is still in its infancy, and it is even unclear whether it will ever reach maturity, the contours of a new paradigm of knowledge production are already becoming visible. We will subsequently sketch the economic, the epistemological and the ethical profile of Do-It-Yourself Bio, and discuss its implications and also its ambivalences.

## Introduction

Since 2008, we witness the emergence of the Do-It-Yourself Biology movement, a global movement spreading the use of biotechnology beyond traditional academic and industrial institutions and into the lay public. Practitioners include a broad mix of amateurs, enthusiasts, students, and trained scientists. According to the movement’s main association - the *DIYbio.org* website – the movement currently counts 26 local groups in Europe, 35 in the United States and Canada, and 11 in Latin America, Asia and Oceania.^1^

The emergence of DIY-Bio followed closely upon the heels of the new scientific discipline of synthetic biology, which had established itself in the early years of the new millennium, after a working draft of the human genome was completed by the Human Genome Project. According to cultural anthropologist Sophia Roosth, who attended both the rise of synthetic biology and the subsequent birth of the first DIY-Bio group in the Boston-Cambridge area as a participant observer, the former actually *spawned* Do-It-Yourself Biology (Roosth 2010, 124).

The most important contribution of the early pioneers to the rise of DIY-Bio was arguably the creation, from 2003 on, of what would become the annual tradition of the *iGEM* contests (international Genetically Engineered Machines), “which has probably done more than any other event to create a generation of biohackers” (Kelty 2010, 4). In these international contests, university student teams compete to make synthetic systems that work in living cells. Several founders of the numerous DIY-Bio groups that emerged after 2008 had been participants at *iGEM* competitions. Students participating in *iGEM* also contribute to the expansion of the Registry of Standard Biological Parts called *BioBricks* – analogous to open-source software registries (Endy 2005).

Already in 2000, some of the synthetic biology pioneers foresaw the rise of an amateur branch of ‘garage biology’ parallel to their own field as a consequence of the ever declining cost curves for DNA sequencing and DNA synthesis (Ledford 2010). In 2001, Robert Carlson predicted that, as these technologies would become less expensive, faster, and ever simpler to use, they would “first move from academic labs and large biotechnology companies to small businesses, and eventually to the home garage and the kitchen” (Carlson 2001, 16). In 2005, he declared: “The era of garage biology is upon us. Want to participate? Take a moment to buy yourself a molecular biology lab on eBay (Carlson 2005). That year, Carlson was the first to build a lab in his garage from equipment bought online.

The DIY-Bio movement has developed under the influence of at least four related movements. First, of course, the do-it-yourself movement that became popular in the 1990s and mainly aimed at home improvement, although DIY biologists already as early as 2009 admitted that ‘do-it-together’ would have been a more appropriate label (Grushkin et al. 2013, 9; Meyer 2014). Second, DIY- Bio is part of the citizen science movement, be it that DIY-Bio projects are not initiated and supervised by scientists within academic institutions like most traditional forms of citizen science but have a genuinely bottom-up character (Ahteensuu and Blockus 2016). Third, DIY-Bio represents a direct translation of free software and hacking practices from the realm of computers and software into the realm of genes en cells (Delfanti 2012, 163). DIY-bio has largely adopted the general principles of the hacker ethic such as sharing, openness, decentralization, free access to computers or tech, and world improvement (Levy 1984). Fourth, DIY-Bio has affinity with the maker movement that represents an expansion of the hacker culture and ethics from software to hardware development.^2^ Apart from traditional activities such as metalworking and woodworking, the maker culture is interested in robotics, 3-D printing and the use of Computer Numeric Control (CNC) tools. Like DIY-Bio the maker movement is a growth area for grassroots entrepreneurship (Anderson 2013, 9).

It is generally accepted that DIY-Bio does not represent new science but a new way of doing science - “a different way of engaging with science and technology, and with the making of things and futures” (Delgado 2013, 66; cf. Wohlsen 2011, 15). The question is therefore whether such overlapping features as transparency and openness, participation and sharing, co-production of experts and lay people, grassroots entrepreneurship et cetera add up to something like a novel, alternative paradigm of knowledge production outside of the academia and industry walls. Our inquiry into this question is organized around three key aspects of DIY-Bio’s new way of doing science, namely its economic, epistemological and ethical aspects.

To examine DIY-Bio’s economic, epistemological and ethical profile, we have reviewed the relevant scientific literature (articles, books, and reports) on the DIY-Bio movement. We have also analyzed documents (manifestos, websites, and codes) from within the movement. In addition, we have made a site visit to the *Waag Society* in the heart of Amsterdam. With over 800 biohackers and a wide range of activities, the *Waag Society* is clearly one of the better DIY-Bio hubs. We also did on-site interviews with the head of the *Waag Society*’s Open Wetlab, Lucas Evers, and with Pieter van Boheemen, Netherlands’ most known DIY-biohacker.

### The economic profile – DIY-Bio versus BIG-Bio

Despite the fact that, mainly through the *iGEM* channel, synthetic biology has been instrumental in the subsequent birth of its non-academic sibling, the two branches of constructive biology remain located at different sides of the institutional divide. As a pursuit performed in million-dollar university labs, academic synthetic biology undoubtedly belongs with its industrial counterpart to BIG-Bio, leaving its younger sibling outside this privileged institutional complex.^3^

BIG-Bio’s research is highly profit-driven. The emphasis is on economic productivity; cells are considered as genetically engineered machines and living factories to create economically viable materials for chemical and pharmaceutical industries and the energy sector. It is estimated that the global market for synthetic biology was US$1.1 billion in 2010. This is expected to increase to $10.8 billion in 2016, with chemicals and energy constituting the largest share (Dana et al. 2012).

DIY-Bio’s business model seems to be the exact opposite of BIG-Bio’s model. The ethics and practices of the DIY-Bio movement are inspired by the hacker movement, with its emphasis on access, sharing, collaboration and decentralization. The movement is organized around ideas of crowd sourcing, peer production, open source software, hardware and data. Biohackers challenge today’s BIG-Bio’s concentration of power and try to open up biology to public participation. Whereas BIG-Bio is responsive to big companies, DIY-Bio is responsive to the community. As citizen science ‘in the making’ (Meyer 2013, 8), DIY-Bio is in search for a fruitful synergy between technology development and community building.

The credo of the DIY-Bio’s open source ethos has been expressed thus: “We reject the popular perception that science is only done in million-dollar university, government, or corporate labs; we assert that the right of freedom of inquiry, to do research and pursue understanding under one’s own direction, is as fundamental a right as that of free speech or freedom of religion” (Patterson 2010).

#### DIY-Bio’s ambivalent relationship with BIG-Bio

But despite its rebellion against the ruling principles of the academic-industrial research complex, DIY-Bio is not entirely free to steer its own course vis-à-vis Big-Bio. For one thing, there is a relationship of dependency concerning cheap, second-hand products for amateurs (Meyer 2013, 14). As a result of the technological accelerations within BIG-Bio business, laboratory equipment quickly becomes out-of-date and is today available at a low cost. It is, moreover, the development of DNA sequencing and synthesis at an industrial scale that was crucial in the advent of both BIG-Bio and DIY-Bio, because it made obtaining a synthesized DNA sequence cheap and fast (Delgado 2013, 68).

DIY-Bio’s relationship with Big-Bio is ambivalent in yet another way. On the one hand biohackers act as rebels who challenge the status quo by advocating free access and sharing; on the other hand they may also act as profiteers who resist external interference from public regulations, corporate interests, or academic institutions, in order to accumulate economic profit as well as personal prestige (Golinelli and Henry 2014). Open source projects are not necessarily anti-capitalist, but may even extend the scope of capitalist exploitation (Delfanti 2011, 52; Delgado 2013, 72).

The combination of rebellion and profitable entrepreneurship can be illustrated by innovators from Silicon Valley, the breeding ground and test bed for internet multinationals such as Facebook, Google, Twitter, Yahoo, Airbnb and Uber. Many of these tech-celebrities have wholeheartedly embraced the libertarian credo of ‘Minimum Government, Maximum Freedom.’ A case in point is Peter Thiel, co-author of the 2014 book *Zero to One* about how to build companies that create new things. He is a co-founder of PayPal and an early investor in Facebook, LinkedIn and Zynga, with a net worth of $1.5 billion, who donated $2.6 million in support of presidential candidate Ron Paul, the ‘intellectual godfather’ of the Tea Party movement.

Recently, Andrew Keen has strongly criticized Silicon Valley’s libertarian ethos in his book *The Internet is Not the Answer*. The net, he argues, was meant to be “power to the people, a platform for equality”: an open, decentralised, democratising technology. Instead, it has handed extraordinary power and wealth to a tiny handful of people, while simultaneously, for the rest of us, compounding and often aggravating existing cultural, social and economic inequalities. Keen portrays the internet as a perfect global platform for free-market capitalism – “it’s a libertarian wet dream. Digital Milton Friedman.”^4^

### The bioluminescence project

A remarkable example of turning community endeavors in DIY-Bio into new business opportunities is provided by the “Bioluminescence Project”, which started in 2011 as a citizen science initiative at the hackerspace *Biocurious* in Sunnyvale, California. Somehow, before long, the biotech-software startup *Genome Compiler Corporation* also got involved. In the first half of 2013, three biohackers affiliated with *Biocurious* as well as with *Genome Compiler* captured the public imagination by conducting a crowd-sourcing campaign to raise money for developing bioluminescent plants that would glow in the dark. The idea looked just cool (the promotional video showed images of glowing trees from the movie Avatar), but it was also presented as a step towards a more sustainable future when streets would no longer be lit by electric lamps but by glowing trees (Kickstarter 2013). Once the project succeeded in reaching its goals, each subscriber would receive envelopes with seeds of the genetically modified plants, to be planted in their own backyards or wherever they liked (in the end the project initiators decided to use seeds of *Arabidopsis* plants instead of trees). The campaign organizers had assured themselves beforehand that all this would be entirely legal, provided that they used a gene gun rather than a bacterial vector for inserting the ‘bioluminescent’ genes into the plants during the final step (Callaway 2013). By June 2013, 6000 hackers had already subscribed, bringing in almost 500.000 dollars to fund the project.

Meanwhile, however, the project had also drawn the attention of anti-biotech and environmental watchdogs like the *ETC. Group* and *Friends of the Earth*. They accused the project managers of setting up an unregulated experiment with the deliberate environmental release of hundreds of thousands specimens of a novel synthetic organism across the United States by cynically exploiting a regulatory loophole and urged them to immediately stop this mischievous adventure. The critics failed however to stop the project in its tracks (at the time of writing, April 2016, the seeds are not yet ready to be shipped to the subscribers). The very identity of DIY-bio as an ‘open’ undertaking has also been put to question. In this case the community group *Biocurious* was used as a launching pad for a commercial project by a startup company intent on helping to create a consumer synthetic biology market. *Genome Compiler* simply considered the various DIY-bio groups in the USA as the incipient nuclei of this emerging market. One commentator, Christina Agapakis, highlighted the apparent paradox that for this company the entire Glowing Plant project was actually about selling a future of *open-source* DIY-Bio and synthetic biology (the DNA designs, the methods used and the parts to be synthesized in the project would all be released open source) in order to promote the widespread use of its *proprietary* genome compiling software (Agapakis 2013).

As a business partner of *Genome Compiler*, the DNA synthesis company *Cambrian Genomics* is also involved in the Glowing Plant project: it will laser print the genetic sequences for bioluminescence designed by the former’s software program (Kera 2014, 33). Its leading young entrepreneur Austen Heinz, sometimes portrayed as a “techno-libertarian”, considers it the mission of his startup company (which in its turn is backed by Peter Thiel and other venture capitalists) to “democratize creation” with as little government regulation as possible. His credo is: “Anyone in the world who has a few dollars can make a creature, and that changes the game. It creates a whole new world” (Heinz quoted in Simons 2015; cf. Lee 2015).

### A European example

In Europe, where the California-style techno-libertarian ethos is much less strong, aspiring entrepreneurs are also less inclined to use existing DIY-Bio community groups as springboards for launching biotech startups: “Few DIY groups in Europe attempt to commercialize their products or skills, but prefer to provide research tools and protocols for the public” (Seyfried et al. 2014). This is not to say that such groups always want to stay aloof from the business world. A very remarkable example of a *rapprochement* between DIY-Bio and BIG-Bio is provided by *Biologigaragen* in Copenhagen and the Danish biotech multinational *Novozymes*, the world’s largest producer of enzymes. Both parties decided in 2014 to collaborate in assay technology development on the basis of open-source principles (Biologigaragen 2014). For the company this is nothing less than a paradigm shift, “because we are not aiming at creating and securing IP here – it is exactly the opposite: We strive to learn and share with everybody”.^5^ This is indeed a clear departure from the strategy of aggressive patenting that is so characteristic of the biotech industry in general and that is also followed by *Novozymes* in other areas. The company nonetheless hopes to learn from the collaboration how they can “accelerate R&D at *Novozymes* by employing smarter and lower cost approaches”. 6

### Epistemological profile - tinkering versus engineering

Inspired by the citizen science movement, DIY-Bio aims at the demystification and democratization of science by opening up science to public participation (Landrain et al. 2013; Meyer 2013). At first sight, one would therefore expect that DIY-Bio fits well into the epistemological profile of post-normal science, a participatory approach that has received much attention in recent years. According to Silvio Funtowicz and Jerome Ravetz, who introduced the notion of post-normal science in 1993, under current conditions of high uncertainties and high decision stakes the puzzle-solving routines of normal science (in the Kuhnian sense) are no longer appropriate. A shift from normal to post-normal science is called for. The most prominent characteristic of post-normal science is the extension of the peer community. With the inclusion of an ever-growing set of stakeholders, “post-normal science can provide a path to the democratization of science” (Funtowicz and Ravetz 1993, 739).

Yet, upon closer inspection, DIY-Bio’s epistemological profile differs from post-normal science in one important respect. The main focus of post-normal science is on the dialogue between science and society, on joint deliberation and decision-making through focus groups, Delphi panels, round tables, consensus conferences et cetera. In contrast, DIY- Bio’s focus is on *practicing* science. DIY-Bio fully subscribes to the first principle of the hacker ethic, as formulated in Steven Levy’s 1984 book *Hackers: Heroes of the Computer Revolution*: “Always yield to the hands-on imperative!” Employing this principle requires free access to the tools of scientific investigation, open information, and knowledge sharing.

The important role of this hands-on imperative is apparent from the following quotation from the *Biopunk Manifesto* by Meredith Patterson, the ‘doyenne’ of DIY-Bio: “Scientific literacy is necessary for a functioning society in the modern age. Scientific literacy is not science education. A person educated in science can understand science; a scientifically literate person can *do* science.” As she has once confessed in an interview, Patterson would like to live in a ‘do-ocracy’; a society where active citizens contribute to the public domain by simply doing things instead of voting, deliberating or negotiating.^7^

#### The savage mind

But what exactly does it mean to *do* science in a DIY-Bio context? According to the hands-on imperative, “hackers believe that essential lessons can be learned about the system – about the world – from taking things apart, seeing how they work, and using this knowledge to create new and interesting things” (Levy 1984, 22). Although in the public perception ‘hacking’ has become a synonym for breaking into computer systems, this quote from Steven Levy makes it clear that hacking is, first and foremost, just another word for tinkering.

The very nature of tinkering can be elucidated with reference to the distinction that the French anthropologist Claude Lévi-Strauss has made in *La Pensée Sauvage* (1962) between two modes of scientific thought: on the one hand the modern-day ‘science of the abstract’ based on ‘domesticated’ modes of thought, and on the other hand the long-traditional ‘science of the concrete’ based on ‘wild’ thought. Whereas abstract science proceeds according to the methods of the engineer, the modus operandi of concrete science is that of the bricoleur or tinkerer. An engineer works according to a preconceived plan, with a precise goal for the desired end, and uses material designed specifically toward that end; the tinkerer, by contrast, works without a clear plan by making creative and resourceful use of whatever materials are at hand to produce new objects that possess some kind of unexpected functionality.

Drawing on *La Pensée Sauvage*, François Jacob, a French biologist and Nobel Prize winner, has compared evolution’s mode of operation with that of a bricoleur. “From an old cycle wheel the bricoleur makes a roulette; from a broken chair the cabinet of a radio. Likewise, evolution makes a wing from a leg or a part of an ear from a piece of jaw” (Jacob 1977, 1164). In a similar vein, the American evolutionary biologist Stephen Jay Gould has claimed that evolution is more a bricoleur than an engineer. He has compared the way evolution works with the recycling market of Nairobi, “where old telephone wire becomes jewelry, tin cans get sawed in half to be used as kerosene lamps, oil drum tops are beaten into large cooking pans, and threadless automobile tires become sturdy sandals” (Gould 1996).

By way of tinkering DIY-Biologists have succeeded in producing inexpensive alternatives to expensive biotechnology equipment, decreasing the costs of setting up a laboratory by a factor of 10 up to 100. The famous story of Kay Aull makes it clear that for biotinkerers “the raw materials of biotech are always just a supermarket away” (Wohlsen 2011, 47). In 2009, Aull set up a lab in her bedroom closet for the price of around 1000 dollars, using tossed-off gear. To distil water, she used a rice cooker and a whiskey tumbler; to separate DNA, she built an electrified box from a picture frame and a plastic box lined with aluminium foil; to be able to see the DNA she used a blue Christmas light. Etcetera… Using these rather basic tools, Aull was able to build a hemochromatosis test. Her father had an advanced case of hemochromatosis, one of the most common hereditary diseases in the U.S., and she wanted to find out if she also carried the mutation. Unfortunately, her self-test that cost a fraction of the commercial DNA test turned out to be positive (*idem*, 9–17).

At the time when she set up her lab, Aull could not confine herself to building her own gear, but had to purchase some specialized laboratory equipment, including a Polymerase Chain Reaction (PCR) machine. This machine uses repeated cycles of heating and cooling to produce millions of copies of a specific DNA sequence in approximately two hours. PCR machines cost about $5000 or more, but Aull managed to find a second-hand one on eBay for $59, an antique model from the 1990s. This situation changed dramatically with the introduction in 2011 of *OpenPCR*, an open source tool developed by Tito Jankowski and Josh Perfetto from the California-based hackerspace for biotech. Interested biohackers can order the *OpenPCR* in the form of a kit containing the different elements online for $600, or download the blueprint free and make their own copy. Another open source design that brought down the costs of setting up wetlabs once again is Cathal Garvey’s *DremelFuge*, a small round disk with slots that hold standard microcentrifuge tubes, designed to fit onto a Dremel rotary tool, which can spin the tubes at high velocities.

#### Tinkering’s transformative impact

Although Kay Aull’s hemochromatosis test is not strictly a scientific break-through, the drastic reduction of costs for biotech equipment certainly has a transformative impact on future education and innovation. By making equipment more affordable, DIY-Bio groups can reach out to the lay public with hands-on training and education that would otherwise be accessible only to university students and those in industry. By increasing access to education and equipment, the DIY-Bio movement facilitates and fosters an open model for innovation, characterized by sourcing and sharing the ‘wisdom of the crowd’, and by decentralized problem solving and distributed creativity.

Because open innovation in the context of DIY-Bio emerges from the ground up, its benefits will come more quickly to those at the bottom. Here, the design and development of low-tech and low-cost equipment and applications is not primarily profit-driven but is guided by the hacker’s principle of ‘world improvement’. The DIY-Bio community promises in particular to be a source of cheaper and simpler solutions for problems of environmental degradation, health care, food safety, and food security.

An early example of a cheap and simple application concerning food safety is the *Melaminometer* that was developed in 2008 by Meredith Patterson, the aforementioned author of the *Biopunk Manifesto*. Patterson succeeded in engineering yogurt bacteria so that they will glow green in the presence of melamine, a toxic chemical found in Chinese infant formula that sickened 300,000 infants in 2008. Her test would cost Chinese families no more than one dollar and be easy to use.

Another example, that attracted broad media attention, is *Amplino*, a mobile malaria testing kit developed in 2012 by Dutch DIY-biologists Wouter Bruins, Jelmer Cnossen, and Pieter van Boheemen from the *Waag Society* (see Introduction). They built their first prototype using the heater element from a hair dryer and some other stuff they picked up at the home improvement store Home Depot for 40 euro. *Amplino* is open-source and much cheaper (less than $250) and easier to use than conventional diagnostic systems. It can detect malaria in less than 40 min by using only a single drop of blood (Landrain et al. 2013).

Bringing such cheap and simple, yet reliable, diagnostic devices to the disease-ridden parts of the developing world, makes local doctors less dependent on the developed world’s pharmaceutical-industrial complex. On the other hand, there is a growing number of DIY-Bio communities in the developing South, especially in Asia, where biotinkering is remarkably close to certain indigenous and traditional practices of knowledge creation, to what Lévi-Strauss has called ‘wild thought’ (Kera 2012, 3).

A good example concerns the very first Indian Scanning Tunneling Microscope (STM), which was built in 1988, seven years after the first one, for which the inventors were awarded a Nobel Prize (Bijker 2013). As Pankaj Sekhsaria has argued, the making of this first Indian STM can be seen as a successful application of what he calls ‘technological Jugaad’. Like bricolage, Jugaad is about looking for new meanings and uses for existing objects by reconfiguring materialities to overcome obstacles and find solutions, a form of innovation that is of vital importance for a society such as India where resources are scarce and access is limited. The close resemblance between both concepts becomes evident when Sekhsaria describes how “[d]iscarded refrigerators, stepper motors from junked computers, tubes from car tyres, bungee chords, Viton rubber tubing, weights from the grocers’ shop, aluminium vessels generally used in the kitchen and bobbins from sewing machines were only some of the components that went into the making of the first prototype and the other probe microscopes that followed” (Sekhsaria 2013, 1155).

#### Co-existence of engineering and bricolage

At first glance there seems to be a stark contrast between DIY-Bio’s methodological approach and that of mainstream synthetic biology. Synthetic biology is regarded by many of its practitioners as an engineering technology based on living systems that has to consider “past lessons from when other engineering disciplines emerged from the natural sciences” (Endy 2005, 450). In other words, synbio is considered as a biological counterpart to chemical, mechanical and electrotechnical engineering. Sophia Roosth speaks in this connection rather graphically of “molecular Taylorism” (Roosth 2013, 160).

A key element in this engineering approach is the reduction or elimination of biological complexity by simplification and decontextualisation. One important option to get rid of complexity is to create a so-called ‘minimal genome’ that can function as a basic biological framework or ‘chassis’ into which new, designed and synthesized segments or sequences that encode novel, economic useful functions can be implemented. Another prominent option of simplifying the design process is the creation of standardized, interchangeable components, the aforementioned *BioBricks*, a kind of genetic Lego Bricks, any two of which can be combined in either order to generate new bricks.

As Sophia Roosth has argued, the contrast between tinkering and engineering is more complex than one would expect. She also compares synthetic biology and DIY-Bio by using Lévi-Strauss’s distinction between ‘engineering’ and ‘*bricolage*’: “While biohackers vocally espouse the principles of synthetic biology, I posit that what they mean by making is altogether different than synthetic biologists: *making* is less about following engineering principles than it is about tinkering and ‘making do’…” (Roosth 2010, 110–111). According to Roosth, the fact that biohackers may on occasion use some of the standard biological parts developed by synthetic biology, by no means implies that they will lose their distinct epistemic profile. She considers this profile to be largely determined by the non-institutional setting outside academia and industry in which biohackers operate and by their efforts to “domesticate” the biological and turn it into something quotidian and mundane. Roosth holds that the DIY-Bio approach “assumes a *bricoleur’s* scavenging and creative use of found or built materials, some of which may be BioBrick parts” (*ibid*., 127). In the end she sees no paradox or contradiction in the coexistence of two contrasting epistemic profiles: “Hobbyist tinkering and industrialized manufacture are two modes of production that are not dialectically opposed in the twinned cultures of synthetic biology and DIY biology” (*ibid*., 128).

Roosth’s judgment may reflect the present state of the art in DIY-Bio, in which biohackers only infrequently “scavenge” the online libraries of BioBrick parts to find useful materials for their projects. But this may change in the near future. The question is whether it is possible to borrow standard biological parts on a larger scale without becoming “infected” with the corresponding epistemic approach. Some authors would give a negative answer to this question. To determine what might be achievable for DIY-Bio in the future, Thomas Landrain and his co-authors look at what is already done by *iGEM* student teams; so they explicitly take *iGEM* (and thus synthetic biology) as a *model* for DIY-Bio (Landrain et al. 2013, 122). This would mean that any coexistence of contrasting epistemic profiles that may occur today is unlikely to endure.

The claimed epistemic contrast between synthetic biology and DIY-Bio can also be nuanced from the other side by examining the methodological style of the former more closely. As Maureen O’Mallley has argued, synthetic biology’s ideal of rational design does not describe its actual practice, in which so-called ‘kludges’ prove to be conspicuously ubiquitous: “Synthetic biology’s design processes always, so far, end up as iterative rounds of trial, error, and pragmatic solutions - sometimes referred to as ‘debugging,’ ‘tweaking,’ ‘retrofitting,’ or ‘parameter tuning’ - to make systems behavior fit design specifications… Rather than exemplifying rational, elegant, and efficient design, many devices work because they are kludges” (O’Malley 2009, 382). Where ‘kludging’ is such a pervasive trait of biological engineering, it would seem that synthetic biology involves much more ‘tinkering’ than its official ideology allows.

O’Malley also points to ‘directed evolution’ as a prominent example of ‘kludging’ in synthetic biology. For some time now, synthetic biologists have recognized that rational design – creating entire bacterial genomes from scratch – is prohibitively difficult because of the vast complexity of living systems, of which we have only limited knowledge. They have discovered evolution as a very powerful design strategy. Under the slogan “let evolution do all the hard work for us” (George Church, quoted in Marchant 2011), synthetic biologists have implemented evolution in the laboratory and have developed various methods of ‘directed evolution’. This strategy merges evolutionary tinkering with rational engineering - nature generates a library of mutants, from which man selects the ones that work and can provide us with desired substances.

### Ethical profile – empowerment versus disenfranchising

Now, let’s turn to the question of the ethical implications of the hands-on imperative. Thus far public discussions on synthetic biology have essentially been about risk issues - about biosecurity and biosafety (Dana et al. 2012) -, with issues about intellectual property and social justice coming second. Professional ethicists also raise questions about the ontological and moral status of biosynthetic organisms: Are they natural or artificial? Do they possess inherent worth or only instrumental value? Isn’t synthetic biology a form of ‘playing God’? (Kaebnick and Murray 2014).

The hands-on approach of DIY-Bio opens up the debate for issues that are hardly or not at all addressed and discussed by the general public or by professional ethicists. Take for example the hands-on workshop ‘Do-It-Yourself Genetics for Dummies’, organized by the *Waag Society* (see Introduction). During this workshop, the participants jointly investigated their own genetic heritage, using the latest methods in genetics. They explored the boundaries of the do-it-yourself movement and discussed the ethical implications of the use of do-it-yourself methods in biotechnology, pharma and healthcare for future society.

During another workshop organized by the *Waag Society* participants were engaged in the production of in vitro meat, that is meat that has been cultivated from stem cells. This workshop generated a lot of ethical questions, Lucas Evers, head of the *Waag Society*’s Open Wetlab told us. In vitro meat or ‘cultured meat’ is not an entirely animal-friendly product yet because of the use of bovine calf serum as a growth medium for stem cells. But if you succeed in replacing this animal medium by a vegetable medium such as algae or fungi, can one then still speak of meat? More generally, is ‘test-tube meat’ a natural product - ‘real meat’ -, or is it artificial, ‘soulless meat’? Will cultured meat alienate us further from nature and from animals? Wouldn’t it be better if we changed our behavior? Does dependence on a technological solution amount to moral laziness?

The use of stem cells of mice to produce in vitro meat during the workshop also raised a lot of questions. So far mice, together with rats, rhesus monkeys and humans are the only species from which pluripotent stem cells have been successfully isolated. Stem cells from farm animals such as cows and pigs tend to quickly change into specialized cells. So, it was discussed if one should try to overcome the ‘yuck factor’ during eating a mouse burger given the problems of factory farming: the mass suffering of animals, the alarming CO2 emissions, and the ever expanding use of land, water and energy. And what about the taboo on cannibalism in the case of meat from human stem cells?

#### Bioart: bioethics in action

Like many DIY-Bio labs, especially in Europe, *Waag Society* is not only focused on synthetic biology but also engage artists and designers to enrich and stimulate ethical deliberations about the potentials and pitfalls of biotechnology. “Art contributes significantly more to the social dialogue about life sciences and biotechnology than scientists realize,” says Lucas Evers. “When art and science meet, unique connections arise. Scientists focus on factual knowledge production, whereas artists give meaning to this knowledge by connecting it to ethics, philosophy and aesthetics.”

An eminent bioartist, who is a master at provoking social debate with humor, satire and performance, is Adam Zaretsky. He is a student of Eduardo Kac, who coined the term ‘bioart’, and who stirred up much controversy when he presented Alba, a genetically engineered fluorescent rabbit. In Zaretsky’s workshops participants have to learn lab techniques such as DNA extraction because he is convinced that one needs to have hands-on experience to be able to do bio-ethics. Zaretsky’s work has been rightly called ‘bioethics in action’ by Joanna Zylinska, author of the 2009 book *Bioethics in the Age of New Media*.^8^

An illustrative example of Zaretsky’s ‘bioethics in action’ is the project he conducted in 2009 during his period as artist in residence at *Waag Society*. The project focused on *BioSolar Cells*, a huge research program in which nine Dutch universities work together with over 30 companies, including Exxon-Mobil, Unilever, and Phillips. In this program scientists look for new ways to use and store solar energy. Fossil fuels are declining rapidly, whereas solar power is freely available in high amounts. Photosynthesis provides a means to collect solar energy and convert it directly into products such as biofuels and hydrogen, but generally only 1 to 2 % of the sunlight is captured as usable energy.

In one research line, the Solar Fish project, scientists investigate possible routes to design organisms that can live from photosynthesis similar to plants, but with higher efficiency. They experiment with injecting zebra fish embryos with chloroplasts in the hope that the fish can produce enough glucose to maintain themselves by the use of sunlight. A next step might concern the creation of larger animals and even humans who could survive on solar power. This would dramatically reduce our ecological footprint and solve the food and energy problem.^9^

With his lectures, exhibitions, workshops and performances Zaretsky has exposed and challenged the justifications for the Solar Fish project and the cultural and political values that underpin them. The most spectacular event took place at *Lowlands*, an annual 3-day music and arts festival, where Zaretsky provoked ethical debate by inviting visitors to inject zebra fish embryos with chloroplasts.^10^ Questions that were discussed among the 2000 participants: Are artists allowed to use such a technique even though they pursue other objectives than scientists? Is this technique not too far-fetched and would it not be much more logical to reduce consumption? What would happen to native species and the global ecology if solar cows, solar pigs, and solar chicken would escape from captivity? Is photosynthetic meat unappetizing to you? Why or why not? What will happen to the global economy if humans become sustainable through sunlight alone? With bodily needs provided simply by lying in a hammock under direct sunlight, who would desire to make something so idiotic as money?

Provocative as a bioartist’s interventions may be, they sometimes tend to promote the type of noncommittal futuristic ethics that has been described as ‘if-then’ ethics (Nordmann 2007). Some of the ethical questions triggered by Zaretsky’s performances clearly belong to this category. The experience may be ‘hands-on’, but it apparently stimulates the imagination to take speculative flights into a remote future. We will try to bring the ethical and political debate on DIY-Bio down to earth again by reconnecting it with the more mundane issues of risk, regulation and intellectual property.

#### The coming era of personalized pets, vegetables and ornaments

The indirect effect of the ‘demystification’ pursued by both bioart and DIY-Bio might well be that the public perception of the ‘risks’ of genetic engineering will change in a direction that is more favorable to biotechnology. If Zaretsky can routinely inject zebra fish embryos with chloroplasts, why then would the biotechnological manipulation of life be objectionable at all? Or as one commentator wrote about the fluorescent rabbit created by Zaretsky’s teacher, Eduardo Kac: “In any case, if one wanted to read Kac’s fluorescent bunny as the next era of personalized pets, what should be our objection? Doesn’t our desire for pets necessarily commit us to their objectification and servitude, even though we might claim they are our companions?” (Miah 2011).

Against this background Freeman Dyson’s speculations on ‘Our Biotech Future’ are extremely pertinent. This article has been called “a founding text of biohacking”, and Dyson himself has been described as the “patron saint” of the biohackers (Wohlsen 2011, 195). His message is precisely the announcement of an imminent era of personalized pets and of personalized vegetables and ornamentals. Dyson holds that the ‘domestication’ of biotechnology in everyday life is now upon us after previous decades have seen the ‘domestication’ of computers, that is, the transition from centralized computers in corporate headquarters and big government departments to decentralized computers in our homes. His informed guess is that “genetic engineering will remain unpopular and controversial so long as it remains a centralized activity in the hands of large corporations” (Dyson 2007). If biotech is to be domesticated and to enter our homes, however, the next step is to become user friendly. The best way to do that, Dyson holds, is to put the tools of genetic engineering into the hands of the numerous gardeners and lovers of pets: “There will be do-it-yourself kits for gardeners who will use genetic engineering to breed new varieties of roses and orchids. Also kits for lovers of pigeons and parrots and lizards and snakes to breed new varieties of pets. Breeders of dogs and cats will have their kits too” (*ibid.*). The younger generation will grow up with computer games and with biotech games. Once biotech becomes accessible to “housewives and children”, it will give rise to “an explosion of diversity of new living creatures, rather than the monoculture crops that the big corporations prefer” (*ibid.*). Finally, Dyson also claims that the rise of ‘green’ technology (based on biology), as distinct from ‘gray’ technology (based on physics and chemistry), offers new prospects to rebalance the relation between the city and the countryside and to put an end to rural poverty.

Many proponents of DIY-Bio seem to have taken a leaf out of Dyson’s article. A case in point is the Glowing Plant project already discussed above. The very idea of bioluminescent plants is reminiscent of Eduardo Kac’s fluorescent rabbit and also of fluorescent zebra fish that are already commercially available. It seems to prepare for a coming era of personalized pets, vegetables and ornamentals. In future the initiators intend to move from glowing *Arabidopsis* plants to glowing roses. Apparently, glowing pets and plants – whether fluorescent or luminescent – appeal to the public imagination as particularly ‘cool’. The avowed mission of Austen Heinz’s *Cambrian Genomics*, one of the companies behind the Glowing Plant project, to “democratize creation” also echoes Dyson’s speculations. Even the idea of do-it-yourself kits has been taken up. The Glowing Plant project’s website envisages the possibility to order at the price of 300 dollars a ‘maker kit’, which “includes the ingredients you need to genetically transform your own plant at home or at a DIY Bio lab like Biocurious or Genspace”.^11^

#### The downside of the hands-on imperative

While DIY-Bio aims to open up science to public participation, the question who or what is the ‘public’ becomes increasingly urgent. Chris Kelty argues that the rise of DIY-Bio, along with patient advocacy groups and the open-source software movement, simultaneously changes what it means to be part of the public: “Being in the public is not passive … but aggressively active, and about knowledge, access, experiment and involvement” (Kelty 2010, 8). Does this mean that you only count when you are actively involved? Fundraising by means of crowd-sourcing also actively engages the public and can be seen as DIY sort of way to finance biohacker projects (Pollack 2013). It is significant that the environmental civil-society organization *ETC. Group* which wanted to stop the Glowing Plant project resorted to the same tactics, thereby committing a fatal error of judgment. They thus mounted their own ‘counter-*Kickstarter*’ crowd-funding campaign dubbed *Kickstopper*. Although the *ETC. Group* had some success when the *Kickstarter* website decided to exclude future projects for genetic technologies, their *Kickstopper* campaign raised far less money than the original crowd-funding campaign of the Glowing Plant project. It may have been unwise for the *ETC.* Group to attempt to beat their opponent on a terrain where the latter had a decisive advantage. The environmental watchdog was not sufficiently aware that they were no longer operating under the old ‘technology-as-conflict’ frame, as in their former campaigns against agricultural biotechnology, but under a new ‘technology-as-gadget’ frame inspired by information technology with an image of ‘coolness’: “with ‘technology as gadget’ the public is seen as a player in the technology’s own team, so to say” (Torgersen and Schmidt 2013, 52). One of the initiators of the Glowing Plant Project, Antony Evans, noted afterwards rather cynically that the project had received much more press coverage, media attention and financial support from subscribers than it would otherwise have had *thanks to* the opposition campaigns of environmental organizations like the *ETC. Group* (Evans 2014).

In a sense, the Glowing Plant Project can be seen as an extreme application of the hands-on imperative in ethics, or Meredith Patterson’s ‘do-ocracy’ – its *reduction ad absurdum*, so to say. If the right to decide about an issue devolves upon the ‘doers’ and the active elements of the public, then this automatically entails a disenfranchising of the less active part of the public and of those who are indirectly affected. Lee Worden explains what is amiss with this very Californian, techno-libertarian ideal of ‘do-ocracy’: “In hacker circles and other Petri dishes for cultural experimentation such as Burning Man, the word ‘do-ocracy’ has become popular. It stands for an ethic of self-organization in which anyone who decides to do something is empowered to do it, and to make the decisions about how to do it… This is a simple, powerful form of practical anarchy that works well for getting things done. However, it doesn’t work well for resolving conflicts between people who want different things to happen; it doesn’t protect people who have less ability to do things because of unequal access to time, or to resources, or unequal physical ability; and it is no help to people who believe that certain things just shouldn’t be done at all” (Worden 2012, 219).

There seems to be no easy solution to the problem of how to involve the public. Current formats of public participation in the governance of emerging technologies emphasize the role of deliberation and dialogue with all potential stakeholders (see the previous section). The results of public engagement exercises, especially when organized from above, are however rather disappointing (Torgersen and Schmidt 2013). Such initiatives can even be sabotaged and boycotted. When in April 2013, a Forum on Synthetic Biology was officially launched in Paris, the meeting was immediately disrupted by protesters wearing monkey-masks who rejected the forum as a “hollow debate” and a “masquerade” (Meyer 2013b). So public deliberation within a broad societal debate is not always an effective alternative to the model of a ‘do-ocracy’ preferred by many biohackers.

Of course, DIY biologists will insist that they comply with all existing rules and regulations – but the initiators of the Glowing Plant project were clever enough to exploit a legal loophole in the patchwork of US regulations. They also complacently declared that the large-scale release of bioluminescent plants would be environmentally innocent, but failed to consult ecologists on this matter. Asked in an interview about whether we should embrace the science-fiction future of a completely bioengineered world, Kyle Taylor, one of the project initiators, answered that he thought we are already living in an engineered natural world (Grushkin 2013). Thus biohackers sometimes do not show much concern for the natural environment. Environmentalists will also be alarmed by the following statement from the *Biopunk Manifesto*: “We reject outright the admonishments of the precautionary principle, which is nothing more than a paternalistic attempt to silence researchers by inspiring fear of the unknown” (Patterson 2010).

If DIY biologists want to use techniques of genetic modification, they will need in most countries a special permission to do so. In Europe, where regulations are rather tight, several DIY-Bio groups have started the process to obtain a certified lab status enabling them to perform such work. The Irish biohacker Cathal Garvey, the inventor of the *Dremelfuge*, has been the first to receive a license that allows him to carry out genetic modification (Seyfried et al. 2014, 550). In general, however, “legislation is absolutely not adapted for proposing amateur-specific licenses” (Landrain et al. 2013, 121). Many biohackers undoubtedly hope that regulations will gradually become more relaxed as DIY-Bio is gaining popularity.

As yet public opposition to GMOs, especially in Europe, might still be a formidable obstacle for such relaxation. The above-mentioned Cathal Garvey speculates, however, that the public’s resistance will largely evaporate once GM crops can be personally designed by ordinary citizens and are no longer the exclusive reserve of big corporations. So his conjecture is that the European public does not so much hate GMOs per se as Monsanto and the other biotech companies that attempt to control the international food supply through patented GM seeds. In this connection Garvey appears to invoke Freeman Dyson’s scenario: “someday, we shall hack our own crops” (SpotOn NYC 2012). As we saw above, Dyson prophesied a rosy future for agriculture and the countryside if only biotechnology could be properly domesticated and decentralized. Dyson’s vision on the future of agriculture had triggered a critical response from the poet, farmer and agricultural publicist Wendell Berry: “How can Mr. Dyson suppose that the rural poor will control the power of biotechnology so as to use it for their own advantage? Has he not heard of the patenting of varieties and genes? Has he not heard of the infamous lawsuit of Monsanto against the Canadian farmer Percy Schmeiser?” (Berry et al. 2007).

In Dyson’s defense, however, it must noted that he was fully aware that the domestication of biotechnology implied that “the rules of Open Source sharing will be extended from the exchange of software to the exchange of genes” (Dyson 2007) – which would obviously be incompatible with patents on genes. So, although Dyson did not elaborate this point, the realization of his vision presumes that the existing legislation on intellectual property can somehow be neutralized, suspended or otherwise be made inoperative. Dyson’s young follower Cathal Garvey is even more aware of the need to create space for DIY-Bio by pushing back the reach of intellectual property law.^12^ This is a formidable political challenge, not just for DIY-Bio but also for the *BioBricks* school in synthetic biology (Nelson 2014). Once the latter moves beyond purely academic pursuits and starts developing economically interesting applications, many insiders expect it will be haunted by lawsuits on alleged patent infringements (Calvert 2012; Kahl and Endy 2013). This also holds for DIY-Bio. It too will presumably only be spared the ordeal of patent litigation as long as it is not becoming too ‘serious’.

But let’s assume for the sake of the argument that the IP specter can indeed be laid to rest. Will Dyson’s vision of a ‘green’ technology that is deployed for the benefit of agriculture and the rural poor then be realized at last? We can get some inkling of what the future might bring by considering a recent DIY-Bio project that was jointly initiated in 2014 by two biohacker community labs in the Bay Area and that was aimed at what is ostensibly an agricultural product: cheese. However, once the project succeeds it will no longer be an agricultural product! The two groups ran a fundraising campaign on the crowdsourcing site *Indiegogo*, where they announced their plan as follows: “Biohackers are engineering baker’s yeast to produce Real Vegan Cheese. No cows needed!” The two groups provided an extensive ethical justification, stressing environmental concerns (greenhouse gases from animal husbandry) and animal welfare concerns (see Team: SF Bay Area DIYbio 2014). Although the goals of the project are laudable, it is also clear that it is not farmer-led and does not have the interests of dairy farmers first and foremost at heart. It does not, therefore, bear out Dyson’s expectation that the domestication of biotechnology will correct the imbalance between urban and rural interests in favor of the latter.

Lee Worden reminds the techno-enthusiasts of California and other developed areas that they should not one-sidedly pursue the liberation of technology, but also take the impacts of their actions on the groups and communities that have little or no say in the decisions that set the directions of research and technological development into account: “Justice requires everyone affected to be included in deliberation, or at least to have a voice. Liberation requires accountability, or at least exposure to the consequences of our choices” (Worden 2012, 218).

### Final remarks

Although the DIY-Bio movement is growing rapidly, it is still in its infancy, while it is even unclear whether it will ever reach adulthood. But the significance of its incipient paradigm of knowledge production is not limited to the DIY-Bio movement. DIY-Bio is not an isolated phenomenon but has emerged as one of many Do-It-Yourself initiatives that we see today within different technological domains (food consumption, repairing and re-using electronic waste *etc.*) in which citizens manifest themselves increasingly as makers, menders, and hackers.

Given its many economic, epistemological and ethical ambivalences, it is an open question whether DIY-Bio will evolve as a genuine alternative for BIG-Bio or whether it will turn out to be perfectly compatible with emerging biocapitalism and the ongoing commodification of all aspects of life. In this context, regional differences play an important role. At this moment we are witnessing a tension between the US model of DIY-Bio, with its orientation to market driven entrepreneurship and personal enhancement on the one hand, and the EU model where open access is coupled with social empowerment and community building on the other hand.

This tension is also reflected in the codes of ethics that were drafted in 2011 by participants of congresses of biohackers from regional groups in North America and Europe. In contradiction with the original intention of the leadership of the global umbrella organization DIYbio.org, these conferences have not resulted in one single code but in two separate codes that show some significant differences (Eggleson 2014). There are differences in the ordering and wording of common items. A case in point concerns the item ‘Peaceful Purposes’. The European code stipulates that “Biotechnology must only be used for peaceful purposes”, whereas the North American code has substituted the unequivocal ‘must’ by the more non-committal ‘should’, making the item an option rather than a requirement. But there are also differences with regard to the content of items. Most significant is the absence in the North American code of the items ‘Community’, ‘Responsibility’, and ‘Accountability’. “These absences, combined with the notion that the use of biotechnology only for peaceful purposes isn’t mandatory, render the North American code a much weaker ethical framework than its European counterpart” (*idem*, 191).

There are, however, still other transatlantic divergences. European and North American biohackers have to operate within distinct regulatory structures: in contrast to North American groups, European groups need to obtain a license in order to carry out genetic engineering experiments. In the US there is a strong focus on biosecurity (including bioterrorism), while in Europe the focus is much more on biosafety. Yet another difference concerns DIY-medicine: whereas North American groups attempt to develop an alternative to the established health care practices, European groups rather focus on helping people in developing countries. And finally, amateur biologists in Europe are more than their North American counterparts focused on collaboration with artists and designers (Seyfried et al. 2014).

The gap between the North American and the European model raises some important questions. Will one of these models take precedence over the other? Or will the tension between the two be resolved in a productive way, creating some sort of balance between grassroots entrepreneurship and social innovation? And how will this tension pan out in other geographic regions such as Asia, Africa, and South America? Will the global DIY-bio community evolve into a more united community or will it continue to be a somewhat heterogeneous ‘scene’ (Wray 2016, 179) These are some of the intriguing questions that merit further investigation.

## Footnotes

1 http://diybio.org/local/ (accessed Jan. 25, 2016).

2 The main principles of the movement include: make, share, give, learn, tool up, participate, support, and change (Hatch 2013).

3 Within the academic-industrial complex of BIG-Bio, however, there is a clear divide between the supporters of open-source approaches (mainly academic synthetic biologists of the *BioBricks* school) and those who pursue IP protection for their novel findings (mainly synbio companies) (Van den Belt 2013; Nelson 2014).

4 https://www.theguardian.com/technology/2015/feb/09/andrew-keen-internet-not-answer-interview (Keen 2015)

5 http://www.novozymes.com/en/innovation/innovation-stories/Pages/open-source-colaboration.aspx (accessed April 2016)

6 *ibid*.

7 The term originated in the US Libertarian party, is currently used by the open source and wiki movement and is also popular among participants of the Burning Man event.

8 Robert Zwijnenberg, professor of Art History in relation to the development of Science & Technology at University Leiden, characterized Zaretsky’s work in a similar vein: “By literally participating hands-on in biotechnological practice, he is able to explore and expose the ethical and aesthetic limits of this practice: the hidden desires, the concerns and the expectations” (Waag Society 2012, 11).

9 http://www.biosolarcells.nl/en/maatschappij/ethiek.html (accessed April 2016).

10 https://www.youtube.com/watch?v=sbXktDeFW_I (accessed April 2016).

11 http://www.glowingplant.com/maker (accessed April 2016)

12 https://biohackspace.org/the-diy-bio-salon-comes-to-london/ (accessed April 2016)
